# Revision of the genus *Ptomaphagus* Hellwig (Coleoptera, Leiodidae, Cholevinae) from the Russian Far East and the Korean Peninsula

**DOI:** 10.3897/zookeys.637.9384

**Published:** 2016-11-28

**Authors:** Cheng-Bin Wang, Jan Růžička, Michel Perreau, Masaaki Nishikawa, Sun-Jae Park

**Affiliations:** 1 Department of Ecology, Faculty of Environmental Sciences, Czech University of Life Sciences Prague, Kamýcká 129, CZ-165 21 Praha 6, Czech Republic; 2 IUT Paris Diderot, Université Paris Diderot case 7139 Sorbonne Paris Cité, 5, rue Thomas Mann, 75205 Paris cedex 13, France; 3 Kashiwagaya 1112–16, Ebina, 243–0402 Japan; 4 National Institute of Biological Resources, Incheon, Republic of Korea

**Keywords:** Leiodidae, Cholevinae, Ptomaphagus, taxonomy, new species, the Russian Far East, the Korean Peninsula

## Abstract

The conundrum of Ptomaphagus (s. str.) sibiricus Jeannel, 1934 (Coleoptera, Leiodidae, Cholevinae, Ptomaphagini) is solved, and it is redescribed and newly recorded in South Korea. A new species is also described from the Russian Far East: Ptomaphagus (s. str.) hayashii **sp. n.** Relevant morphological characters of the concerned species are illustrated with colour plates, and their known distributions are mapped.

## Introduction

*Ptomaphagus* Hellwig, 1795 is the most speciose genus (including 137 known species worldwide) in the tribe Ptomaphagini (Coleoptera, Leiodidae, Cholevinae). However, the nominotypical subgenus, which is limited to the Palaearctic and north Oriental regions has only 29 species (Perreau 2000, Nishikawa 2011, Wang et al. 2016a, 2016b).

In the fauna of the Russian Far East, only one species in the subgenus Ptomaphagus s. str. had been recorded before this study, namely Ptomaphagus (s. str.) sibiricus Jeannel, 1934.

However, when we examined specimens previously identified as Ptomaphagus (s. str.) sibiricus from various collections, we discovered that three species were identified under this name by different authors. One of them with conspicuous differences from Japan was already described in a previous paper: Ptomaphagus (s. str.) piccoloi Wang, Růžička, Nishikawa, Perreau & Hayashi, 2016 (Wang et al. 2016a). In this paper, we solve the conundrum of Ptomaphagus (s. str.) sibiricus, and redescribe it and report it for the first time in South Korea. The third species from the Russian Far East is also new, and is described and illustrated here: Ptomaphagus (s. str.) hayashii sp. n. Relevant morphological characters of examined species of *Ptomaphagus* are illustrated with colour plates, and their known distributions are mapped.

## Material and methods

Specimens were relaxed and softened in a hot saturated solution of potassium hydroxide for 4 minutes (for mounted dry specimens) or 8 minutes (for alcohol-preserved specimens), and then transferred to distilled water to rinse the residual potassium hydroxide off and stop any further bleaching. The softened specimens were moved into glycerin and dissected there to observe morphological details. After examination, the body parts were mounted on a glass slip with Euparal Mounting Medium for future studies. Habitus photographs were taken using a Canon macro photo lens MP-E 65mm on a Canon 550D. Observations, photographs and measurements of morphological details were performed using an Olympus BX53 microscope with an Olympus DP73. The final deep focus images were created with Zerene Stacker 1.04 stacking software. Adobe Photoshop CS6 was used for post processing. Exact label data are cited for specimens examined. Authors’ remarks and addenda are placed in square brackets, separate label lines are indicated by a slash (/), and separate labels by a double slash (//). Measurements are averaged over 5 specimens.

The material examined for this study is deposited in the following collections and museums:

BMNH
Natural History Museum (formerly British Museum), London, United Kingdom (M. Barclay) 


CCBW
 Collection of Cheng-Bin Wang, Chengdu, Sichuan, China 


CJRZ
 Collection of Jan Růžička, Prague, Czech Republic 


CMNE
 Collection of Masaaki Nishikawa, Ebina, Japan 


CNUIC
 Chungnam National University Insect Collection, Daejeon, Korea 


MNHN
Muséum National d’Histoire Naturelle, France, Paris (T. Deuve, Azadeh Taghavian) 


NSMT
National Museum of Nature and Science, Tsukuba, Japan (S. Nomura) 


SDEI
Senckenberg Deutsches Entomologisches Institut, Müncheberg, Germany (L. Behne) 


The following abbreviations are used for the measurements in millimetres (mm):

AL
 (antennal length) : length from the antennal base to apex.

BTW
 (basitarsal width) : maximum width of 1st protarsomere.

EBL
 (extended body length) : summation of HL, PL, ELL and length of exposed scutellum, preventing the error introduced by exposed or retracted head.

ELL
 (elytral length) : length from the tail end of scutellum to the elytral apex.

ELW
 (elytral width) : maximum width of the widest portion of two elytra combined together.

EW
 (eye width) : width of a single compound eye in dorsal view.

HL
 (head length) : axial length from the anterior apex of clypeus through the posterior margin of occipital carina.

HW
 (head width) : width of the widest portion of head (usually including eyes).

PL
 (pronotal length) : axial length of the pronotum.

PW
 (pronotal width) : maximum width of pronotum.

TW
 (tibial width) : maximum width of protibia (excluding spines along outer margin etc.).

## Results

### Genus Ptomaphagus

Taxon classificationAnimaliaColeopteraLeiodidae

Hellwig, 1795

#### Distribution.

Holarctic, north Oriental, north Neotropical.

### Subgenus Ptomaphagus s. str.

Taxon classificationAnimaliaColeopteraLeiodidae

#### Distribution.

Palaearctic, north Oriental.

### Ptomaphagus (s. str.) hayashii sp. n.

Taxon classificationAnimaliaColeopteraLeiodidae

http://zoobank.org/C468947A-D211-4411-B09B-775DFF6A7045

Figs 1B, C ; 2 ; 3

#### Type material.

**Holotype**: 1♂, USSR: Sadgorod [ca. 43°15'N 132°03'E] / (in forest; trap with / bait), Vladivostok // Primorskyi Kray / 16.VI.1978 / E. Berlov leg. (NSMT). **Paratypes**: 1♂1♀, FE.Russia, SW Khabarovsky / kray reg., Strel’nikova / range Mts., 46°43'N 134°08'E / Samur Riv., 130–550 m alt. / 25.VI.–28.VII.2014 / A. Plutenko leg. (CMNE); 1♀, same data as holotype (CMNE); 1♀, USSR Ussuri reg. / NOVOVARVAROVKA [ca. 43°58'N 132°59'E] / 6–10.VII.1989 / R. Červenka lgt. // Ptomaphagus (s. str.) / sibiricus / Jeannel, 1934 / Jan Růžička det. 2001 // ♀ (CJRZ); 1♂1♀, USSR Ussuri reg. / JASNOE [= Yasnoye, ca. 43°27'N 132°09'E] VII.1989 / St. Bečvář lgt. // Ptomaphagus (s. str.) / sibiricus / Jeannel, 1934 / Jan Růžička det. 2001 (CJRZ); 1♂1♀, USSR Ussuri reg. / JASNOE [= Yasnoye] / VII.1989 12–16 / St. Bečvář lgt. // MOUNT. OBLATCHNAJA [= Oblachnaya] / 400–800 m (CJRZ); 1♀, RUSSIA, Far East: / S Primorye region, / LAZO env. [ca. 43°22'N 133°54'E], 2.VII. / 2002 // Ptomaphagus (s. str.) / sibiricus / Jeannel, 1934 / Jan Růžička det 2009 / ♀ (CJRZ); 1♂, RUSSIA: Primorsky / 30 km NE Vladivostok / Tajvaza / 29.VII–5.VIII.1992 / B. D. Gill [leg.] (CJRZ); 1♂, Russian Far East, Primorskii krai / Lazovski Zapovednik, c. 170 km E / Vladivostok, Korpad, 1.–14.v.2001 / 175 m; 43°15'47"N 134°07'44"E / floodplain of Priamushka / Malaise trap 440; / M. Quest coll. BMNH (E) 2009-59 (BMNH); 1♀, same data as previous except: 13.–31.vii.2001 / 43°15'52"N 134°07'45"E; 174 m; / Mountain top, Malaise trap 481 (BMNH).

#### Description.

*Male*. EBL: 3.8–4.3 mm (4.2 mm in holotype). Length of different body parts: HL : AL : PL : ELL = 0.7 : 1.1 : 1.1 : 2.3 mm; width: HW : EW : PW : ELW = 1.1 : 0.1 : 1.6 : 1.8 mm. Proportion of antennomeres from base to tip in μm (length × width): 149 × 75, 109 × 67, 95 × 70, 64 × 79, 58 × 92, 41 × 100, 91 × 128, 39 × 128, 87 × 151, 90 × 156, 161 × 143.

**Figure 1.**
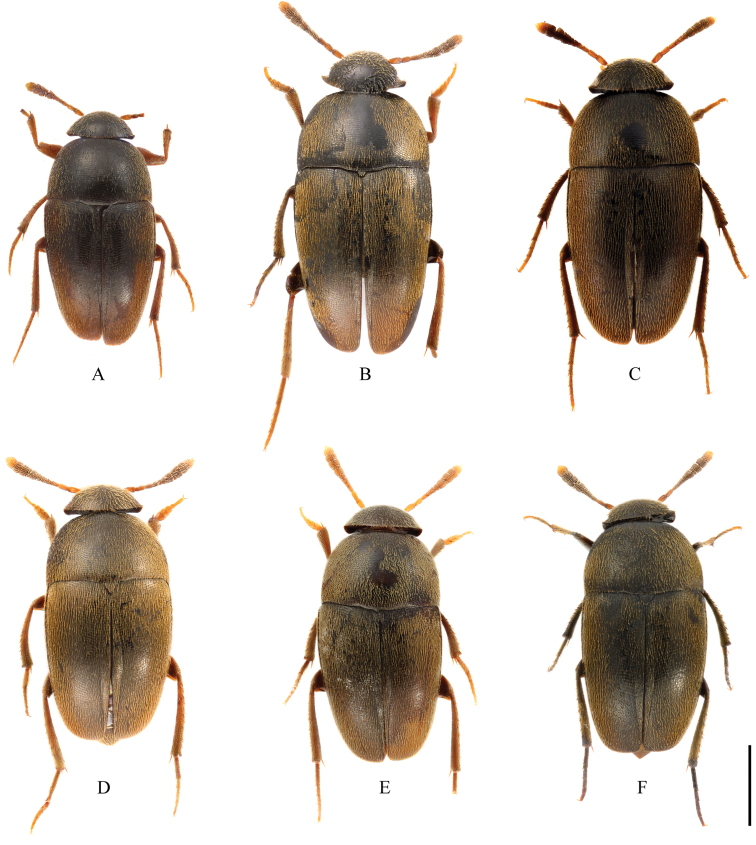
Habitus of *Ptomaphagus* (s. str.) spp. (dorsal view). **A, D–F** *Ptomaphagus* (s. str.) *sibiricus* Jeannel, 1934 **A** ♀ (holotype; Vladivostok) **D** ♂ (Vladivostok) **E** ♂ (Pyeongchang-gun) **F** ♀ (Boeun-gun) **B, C** *Ptomaphagus* (s. str.) *hayashii* sp. n. **B** ♂ (holotype; Vladivostok) **C** ♀ (paratype; Ussuri region). Scale: 1 mm.

Habitus (Fig. 1B) elongated oval, regularly convex and sublustrous. Well pigmented: mostly blackish brown; mouthparts, antennae (apical half of ultimate antennomere yellowish) and tarsi reddish brown. Dorsum continuously clothed with fine, recumbent, yellowish pubescence. Insertions of pubescence on dorsal surfaces of pronotum, elytra and femora aligned along transverse striolations; interspace between two striolations glabrous.

**Figure 2.**
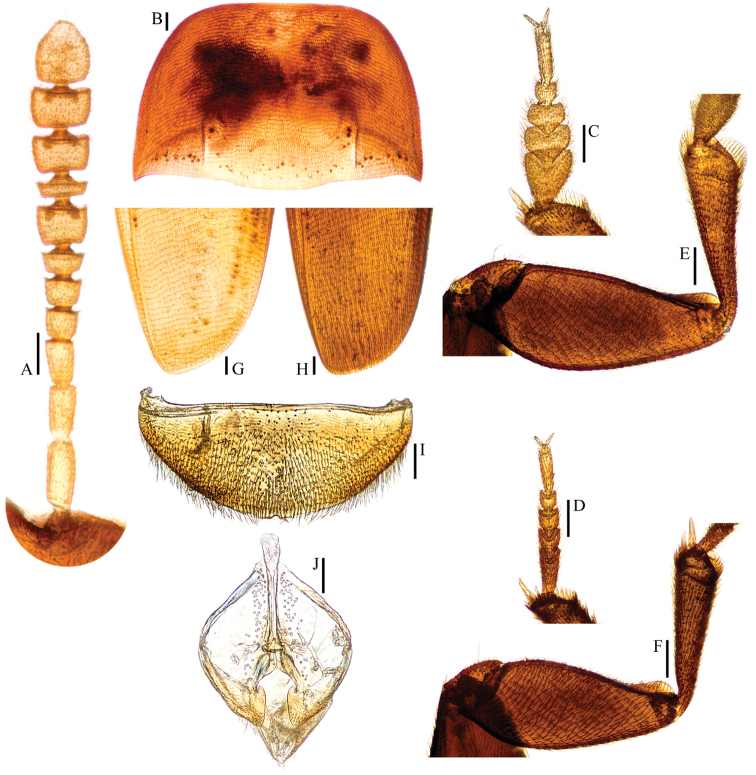
*Ptomaphagus* (s. str.) *hayashii* sp. n. (♂: holotype). **A** antenna ♂ (dorsal view) **B** pronotum ♂ (dorsal view) **C** protarsus ♂ (dorsal view) **D** protarsus ♀ (dorsal view) **E** protibia and profemur ♂ (ventral view) **F** protibia and profemur ♀ (ventral view) **G** elytral apex ♂ (dorsoapical view) **H** elytral apex ♀ (dorsoapical view) **I** ventrite VIII ♂ (ventral view) **J** genital segment ♂ (ventral view). Scales: 0.1 mm.

Head transverse, HW/HL = 1.6. Clypeofrontal suture absent. Clypeus with anterior margin slightly rounded. Compound eyes well developed, EW/HW = 0.11. Antennae (Fig. 2A) slender, AL/HW = 1.0; antennomere III shorter than II; VI with length/width = 0.4; XI peach-shape.

Pronotum (Fig. 2B) transverse, widest just before hind angles, PW/PL = 1.5. Sides gently arched, simply narrowing from posterior to anterior; hind angles slightly projected backwards and acute. Posterior margin widely protruded in middle part, emarginate near hind angles.

Elytra oval, widest at about basal 1/4, ELL/EW = 1.3. Sides weakly arched, gradually narrowing from widest part to apices; apices (Fig. 2G) narrowly rounded. Sutural striae present. Metathoracic wings fully developed.

Prolegs robust, with basal three protarsomeres (Fig. 2C) strongly expanded: TW/BTW = 1.2. Protibiae (Fig. 2E) strongly expanded towards apex. Profemora broad. Mesotibiae arcuate, mesotarsi simply linear. Metatibiae slender and straight.

Abdominal ventrite VIII (Fig. 2I) round at posterior edge and with an inconspicuous median notch. Spiculum gastrale (Fig. 2J) of genital segment with about 1/5 of length protruding beyond anterior edge of epipleurite IX.

**Figure 3.**
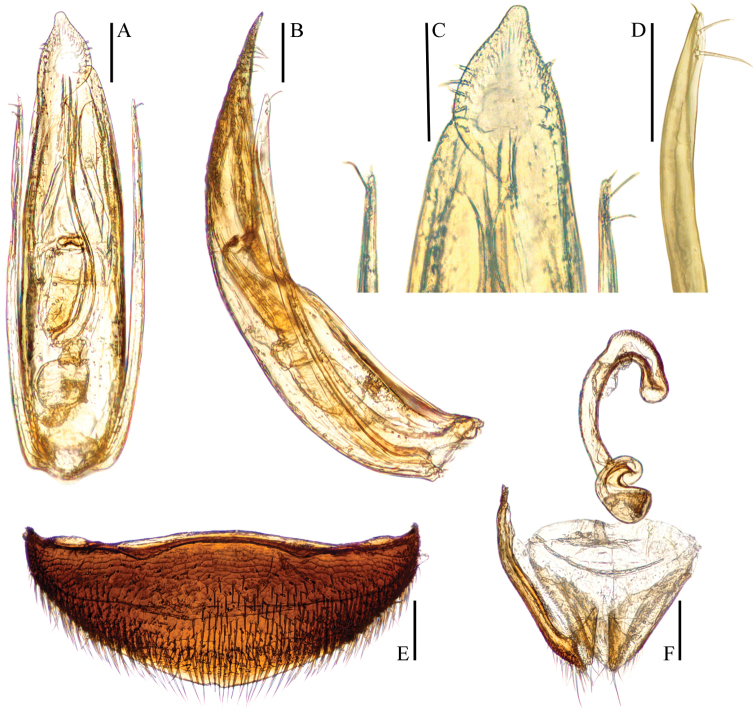
*Ptomaphagus* (s. str.) *hayashii* sp. n. (♂: holotype). **A** aedeagus (dorsal view) **B** aedeagus (lateral view) **C** aedeagal apex (ventral view) **D** paramere apex (lateral view) **E** ventrite VIII ♀ (ventral view) **F** spermatheca, genital segment and ovipositor (ventral view). Scales: 0.1 mm.

Aedeagus (Fig. 3A) long and slender, with median lobe gradually narrowing towards a lanceolate apex and terminated to a widely rounded knob in dorsal view; opening of genital orifice situated on dorsal surface, deeply cut inwards on preapical left margin of median lobe. Ventral surface of the apex of the median lobe (Fig. 3C) inserted with 5 ventrally oriented setae on both sides; parameres narrow, reaching about apical 1/5 of median lobe, each apex (Fig. 3D) with 2 lateral setae and 1 apical seta distinctly shorter. In lateral view (Fig. 3B), median lobe regularly bent ventrad and gradually tapering to a flat apex. Endophallus with stylus quite slender, a subelliptical nodule in middle region, a cheliform complex just below base of stylus, and a circular complex in the basal region.

*Female*. Similar to male in general appearance (Fig. 1C), including elytral apices (Fig. 2H), but distinguished by the following characteristics: protarsi (Fig. 2D) simply linear; protibiae (Fig. 2F) narrower; abdominal ventrite VIII (Fig. 3E) narrowly rounded at posterior edge; genital segment and ovipositor as shown in Fig. 3F; spermatheca (Fig. 3F) curved in distal part and coiled in proximal part.

#### Diagnosis.

*Ptomaphagus* (s. str.) *sibiricus* is sympatric with *Ptomaphagus* (s. str.) *hayashii* sp. n. in some locations of the Russian Far East, and they are very similar to each other (including spermatheca (Figs 3F; 5F), which is only more roundly curved in the stem part in *Ptomaphagus* (s. str.) *hayashii* sp. n.). For external characters, the body size of *Ptomaphagus* (s. str.) *sibiricus* (Figs 1A, D–F) is a little smaller than *Ptomaphagus* (s. str.) *hayashii* sp. n. (Figs 1B, C). However, their aedeagi provide critical characters to distinguish the two species: in *Ptomaphagus* (s. str.) *hayashii* sp. n., the aedeagus is much larger and more slender (Fig. 3A), the right apicoventral piece of the median lobe is slenderly lanceolate (Fig. 3C), the apical half of median lobe much flatter in lateral view (Fig. 3B); while in *Ptomaphagus* (s. str.) *sibiricus*, the aedeagus is stouter (Fig. 5A), the right apicoventral piece of median lobe is much wider and subpentagonal (Fig. 5C), the apical half of the median lobe thicker in lateral view (Fig. 5B).

**Figure 4.**
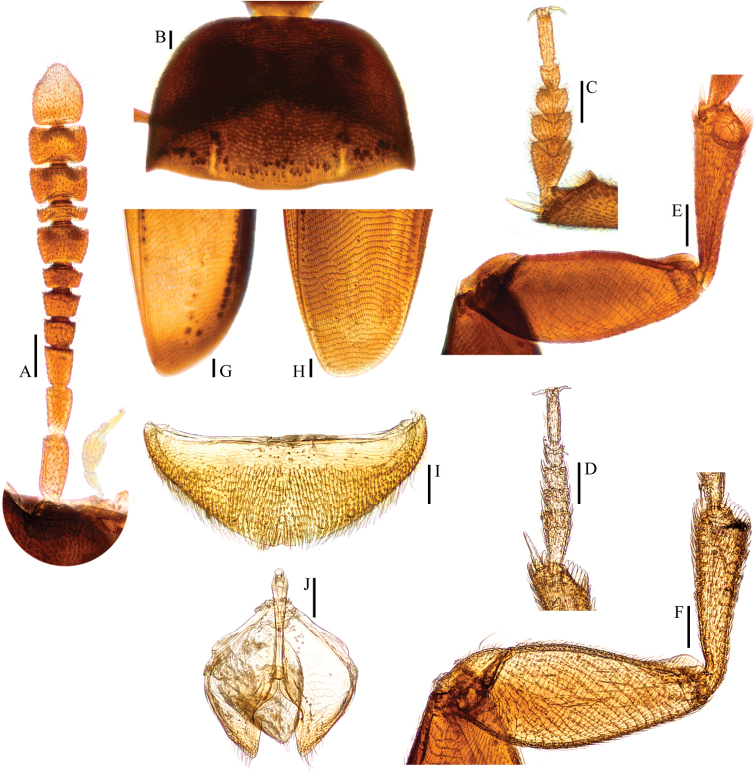
*Ptomaphagus* (s. str.) *sibiricus* Jeannel, 1934 (Vladivostok). **A** antenna ♂ (dorsal view) **B** pronotum ♂ (dorsal view) **C** protarsus ♂ (dorsal view) **D** protarsus ♀ (dorsal view) **E** protibia and profemur ♂ (ventral view) **F** protibia and profemur ♀ (ventral view) **G** elytral apex ♂ (dorsoapical view) **H** elytral apex ♀ (dorsoapical view) **I** ventrite VIII ♂ (ventral view) **J** genital segment ♂ (ventral view). Scales: 0.1 mm.

**Figure 5.**
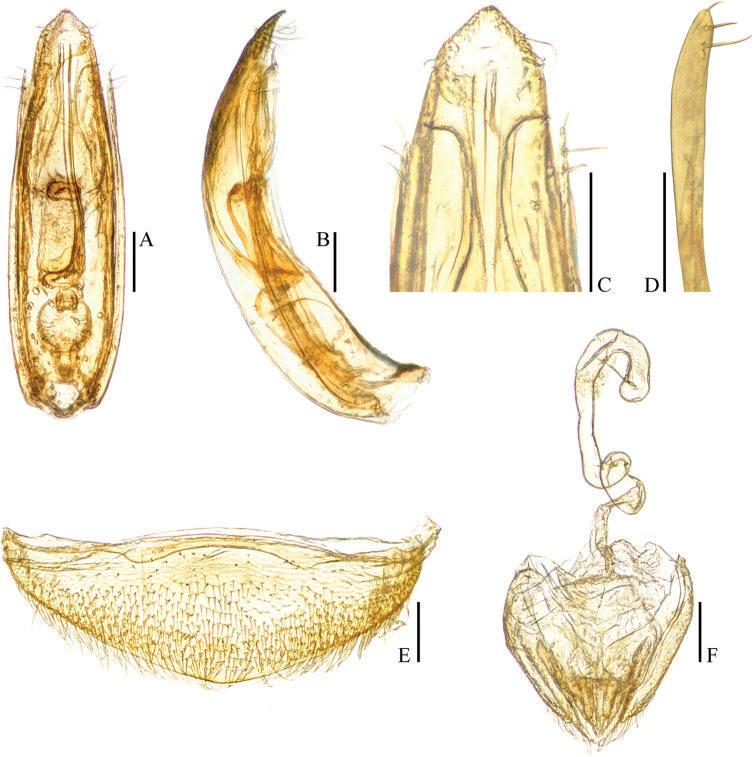
*Ptomaphagus* (s. str.) *sibiricus* Jeannel, 1934 (Vladivostok). **A** aedeagus (dorsal view) **B** aedeagus (lateral view) **C** aedeagal apex (ventral view) **D** paramere apex (lateral view) **E** ventrite VIII ♀ (ventral view) **F** spermatheca, genital segment and ovipositor (ventral view). Scales: 0.1 mm.

Furthermore, based on specimens examined, *Ptomaphagus* (s. str.) *sibiricus* seems to be much more widely distributed, extending southward to South Korea; while *Ptomaphagus* (s. str.) *hayashii* sp. n. is presently known only in the Russian Far East.

#### Etymology.

The specific epithet is dedicated to Mr. Yasuhiko Hayashi (Kawanishi, Japan), a famous independent taxonomist on Staphylinoidea, for his continual generous help to our study.

#### Distribution.

**Figure 6.**
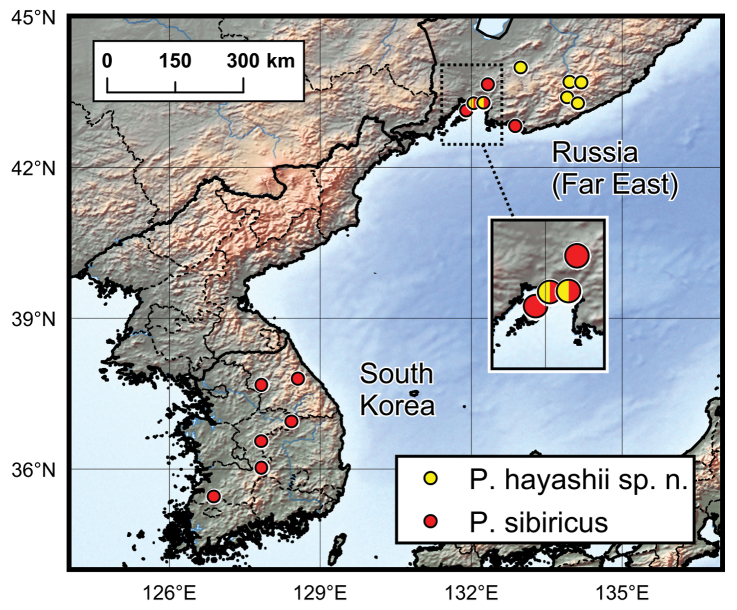
Distribution map of *Ptomaphagus* species from the Russian Far East and the Korean Peninsula.

Russia (Far East) (Fig. 6).

### Ptomaphagus (s. str.) sibiricus

Taxon classificationAnimaliaColeopteraLeiodidae

Jeannel, 1934

Figs 1A, D–F ; 4 ; 5

Ptomaphagus (s. str.) sibiricus Jeannel 1934: 165 (Ptomaphagus (s. str.); type locality: [RUSSIA, Far East] Wladiwostok; SDEI); Jeannel 1936: 72, 84 (Ptomaphagus (s. str.); in key; distribution); Nishikawa 1983: 1 (Ptomaphagus (Ptomaphagus); in check-list); Perreau 2000: 364 (Ptomaphagus (s. str.); in catalogue); Perreau 2004: 178 (Ptomaphagus (Ptomaphagus); in catalogue); Zinchenko and Lyubechanskii 2008: 340 (Ptomaphagus; distribution); Perreau 2015: 249 (Ptomaphagus (Ptomaphagus); in catalogue).

#### Material examined.

**Type material.** **Holotype:** ♀, [RUSSIA, Far East] Wladiwostok [ca. 43°10'N 132°00'E] // Reitter // Coll. Koltze // Pt. variicornis / Rosenh. // Ptomaphagus sibiricus Jeann. / type / R. Jeannel det. // DEI Müncheberg / Col – 07069 (SDEI).

**Additional material.** **RUSSIA, Far East:** 1♀, FE.Russia, SW Khabarovsky / kray reg., Strel’nikova / range Mts., 46°43'N 134°08'E / Samur Riv., 130–550 m alt. / 25.VI.–28.VII.2014 / A. Plutenko leg. (CMNE); 1♀, Vladivostok [ca. 43°10'N 132°00'E] / Christov [leg.] IX.[18]76 // Ptomaphagus sibiricus / Jeannel det. // Ptomaphagus / (Ptomaphagus) sibiricus / Jeannel, 1934 / Ex. M. Nishikawa, 2008 / # MNHN 103377Ch1S ♀ (MNHN); 10♂♂10♀♀, RUSSIA: Primorsky / 30 km NE Vladivostok / Tajvaza / 29.VII–5.VIII.1992 / B. D. Gill [leg.] (CJRZ); 1♀, RUSSIA: Primoryi / Nakhodka [ca. 42°49'N 132°52'E] / 6-8.VIII.1999 / B. D. Gill [leg.] (CJRZ); 1♂, USSR: Sadgorod [ca. 43°15'N 132°03'E]/ (in forest; trap with / bait), Vladivostok // Primorskyi Kray / 16.VI.1978 / E. Berlov leg. (CMNE); 1♂, Primorskiy Kray, Ussuriyskiy Region, Uss. [uriyskiy] Zapov. [ednik, = Reserve], Staraya Baza, 43.64°N, 132.34°E, poch.l. [= pitfall trap], V. Zinchenko & A. Korshunov [leg.], 9.–19.viii.2011 // Ptomaphagus / sibiricus / V. Zinchenko det. 2002 (CCBW). **South Korea**: 1♂, KOREA: Gangwon Prov., / Pyeongchang-gun, Jinbu-myeon, / Dongsan-ri, Mt. Odaesan, Sangwonsa [ca. 37°47'N 128°33'E], / 4.VI.–22.VI.2001, K.-J. Ahn, S.-J. / Park, M.-S. Kim, M.-J. Jeon [leg.], ex FIT // Ptomaphagus / baekamsanensis / n. sp. / det. S.-J. Park 2005 // 1 (CNUIC); 1♂, KOREA: Gangwon Prov., / Hongcheon-gun [ca. 37°41'N 127°50'E], Mt. Baekamsan, / 25.V.–20.VI.2002, K J Ahn, / C W Shin, J S Park, ex FIT // Ptomaphagus koreanus / new species / det. Sun-Jae Park (CNUIC); 1♀, KOREA, Chungbuk Prov., / Danyanggun, Youngchunmyeon, / Namcheonri, Namcheon valley, / Mt. Sobaeksan [ca. 36°57'N 128°26'E], (M.T), / 25.V.–6.VII.2006 (CNUIC); 1♂, same data as previous except: 2006.VII.6–VII.28 (CNUIC); 1♀, Korea, Kyungsangbuk-do / Yuongdong-gun / Sangchon-myun, Mulhan-ri / Mt. Minjujisan [ca. 36°02'N 127°50'E] / VI.16–18.2000, / Y.B. Cho / coll. / ex bait trap // ♀ No.9 (CNUIC); 1♀, same data as previous except: ♀ 10 (CNUIC); 4♂♂1♀, Korea, Chonrabukdo / Selchonmyun / Mt. Deokyusan / Baekyeonsa Temple [ca. 35°26'N 126°52'E] // vi.16.1999 / D. S. Kim coll. / ex bait trap // Ptomaphagus / sp. / Det. M. Nishikawa // [5, 6, 13, 14 & 15, respectively] (CNUIC); 1♂2♀♀, KOREA: Chungbuk Prov., / Boeun-gun, Mt. Sokrisan [ca. 36°32'N 127°54'E], / Bubjusa, 2004.VI.1–28, / Y.-B. Cho [leg.], FIT (CMNE).

#### Redescription.

*Male*. EBL: 3.1–3.8 mm (3.1 mm in holotype). Length of different body parts: HL : AL : PL : ELL = 0.6 : 1.0 : 1.0 : 1.8 mm; width: HW : EW : PW : ELW = 1.0 : 0.1 : 1.5 : 1.6 mm. Proportion of antennomeres from base to tip in μm (length × width): 134 × 66, 107 × 65, 77 × 67, 55 × 81, 54 × 88, 47 × 102, 88 × 125, 35 × 128, 82 × 140, 82 × 140, 147 × 127.

Habitus (Figs 1D, E) elongated oval, regularly convex and sublustrous. Well pigmented: mostly dark brown; mouthparts, basal three antennomeres and apical half of ultimate antennomere, protarsi, and apex of meso- and metatarsi more or less paler. Dorsum continuously clothed with fine, recumbent, yellowish pubescence. Insertions of pubescence on dorsal surfaces of pronotum, elytra and femora align along transverse striolations; interspace between two striolations glabrous.

Head quite transverse, HW/HL = 1.7. Clypeofrontal suture absent. Clypeus with anterior margin almost straight. Compound eyes well developed, EW/HW = 0.1. Antennae (Fig. 4A) slender, AL/HW = 1.0; antennomere III shorter than II; VI with length/width = 0.5; XI peach-shape.

Pronotum (Fig. 4B) transverse, widest just before hind angles, PW/PL = 1.4. Sides gently arched, gradually narrowing from posterior to anterior; hind angles projected backwards and acute. Posterior margin widely protruded in middle part, distinctly emarginate near hind angles.

Elytra oval, widest at about basal 1/3, ELL/EW = 1.2. Sides weakly arched, gradually narrowing from widest part to apices; apices (Fig. 4G) narrowly to widely rounded (all examined specimens from South Korea with wide elytral apices). Sutural striae present. Metathoracic wings fully developed.

Prolegs robust, with basal three protarsomeres (Fig. 4C) strongly expanded: TW/BTW = 1.3. Protibiae (Fig. 4E) distinctly expanded towards apex. Profemora broad. Mesotibiae arcuate, mesotarsi simply linear. Metatibiae slender and straight.

Abdominal ventrite VIII (Fig. 4I) rounded and with an inconspicuous median notch at posterior edge. Spiculum gastrale (Fig. 4J) of genital segment with about 1/5 of length protruding beyond anterior edge of epipleurite IX.

Aedeagus (Fig. 5A) long, slender but relatively strong, with median lobe gradually narrowing towards a wide subpentagonal apex and terminated to a widely rounded knob in dorsal view; opening of genital orifice situated on dorsal surface, deeply cut inwards on preapical left margin of median lobe. Ventral surface of the apex of the median lobe (Fig. 5C) inserted with 6 ventrally oriented setae on the left side and 5 ventrally oriented setae on the rigth side; parameres narrow, reaching about apical 1/6 of median lobe, each apex (Fig. 5D) with 2 lateral setae and 1 apical seta distinctly shorter. In lateral view (Fig. 5B), median lobe thick, regularly bent ventrad, and gradually tapering to a thin apex. Endophallus with stylus quite slender, a cheliform complex just below base of stylus, and a circular complex in the basal region.

*Female*. Similar to male in general appearance (Fig. 1A, F), including elytral apices (Fig. 4H) and protibiae (Fig. 4F), but distinguished by the following characteristics: protarsi (Fig. 4D) simply linear; abdominal ventrite VIII (Fig. 5E) rounded at posterior edge; genital segment and ovipositor as shown in Fig. 5F; spermatheca (Fig. 5F) curved in distal part and coiled in proximal part.

#### Diagnosis.

See under *Ptomaphagus* (s. str.) *hayashii* sp. n. above. Other remarks on this species see Wang et al. (2016a).

#### Distribution.

Russia (Far East), South Korea (Fig. 6).

## Supplementary Material

XML Treatment for Ptomaphagus

XML Treatment for Ptomaphagus s. str.

XML Treatment for Ptomaphagus (s. str.) hayashii

XML Treatment for Ptomaphagus (s. str.) sibiricus
